# Once-daily tigecycline for outpatient parenteral antibiotic therapy: a single-centre observational study

**DOI:** 10.1093/jacamr/dlz085

**Published:** 2019-12-24

**Authors:** Stephen Hughes, Hui Yin Chin, Katie L Heard, Pegah Kamranpour, Brent Bartholomew, Nabeela Mughal, Luke S P Moore

**Affiliations:** 1 Chelsea and Westminster NHS Foundation Trust, 369 Fulham Road, London SW10 9NH, UK; 2 North West London Pathology, Imperial College Healthcare NHS Trust, Fulham Palace Road, London W6 8RF, UK; 3 National Institute for Health Research Health Protection Research Unit in Healthcare Associated Infections and Antimicrobial Resistance, Imperial College London, Hammersmith Campus, Du Cane Road, London W12 0NN, UK

## Abstract

**Background:**

Tigecycline has potential utility in the treatment of complex polymicrobial infections or those caused by MDR organisms in the ambulatory care setting owing to its breadth of antimicrobial coverage. Whilst licensed for twice-daily IV administration, its long half-life permits once-daily administration, which may facilitate successful outpatient parenteral antibiotic therapy (OPAT).

**Methods:**

A retrospective case series of patients receiving once-daily tigecycline under OPAT was analysed at a single-centre NHS acute hospital (January 2016–June 2018). Patient demographics, including comorbidities, antimicrobial indication, concurrent antimicrobial therapies, treatment duration and adverse events related to treatment were recorded using medical records. Treatment outcomes were defined using the BSAC National Outcomes Registry System (NORS).

**Results:**

A total of 25 treatment episodes (24 individual patients) were analysed. The most common indications were bone and joint infections (*n *=* *8) and intra-abdominal infections (*n *=* *7). MDR organisms were common, including ESBL-producing Enterobacterales (*n *=* *13) and glycopeptide-resistant enterococci (*n *=* *4). Median treatment duration was 18 days. Nineteen of 25 (76%) cases had complete cure of treatment, 3 patients experienced treatment-related adverse reactions necessitating cessation of therapy and 3 experienced failure due to disease progression. Eight patients experienced non-limiting adverse effects, such as nausea, vomiting and rash, and one patient had a transient rise in amylase 3 times the upper normal limit (with no evidence of pancreatitis).

**Conclusions:**

Once-daily tigecycline can be successfully used for management of complex infections in the OPAT setting, with predominantly mild adverse effects, which can be managed with antiemetics or slow administration.

## Introduction

Tigecycline is a glycylcycline with activity against a range of Gram-positive and Gram-negative organisms including MRSA, glycopeptide-resistant *Enterococcus* spp. (GRE), ESBL-producing Enterobacterales and some carbapenem-resistant organisms (CROs).^1^ Tigecycline is therefore increasingly being used to treat complicated skin and soft tissue infections, intra-abdominal infections and, off-label, bone and joint infections, typically where resistant organisms or polymicrobial infections are identified.^2–4^ Concerns about tigecycline efficacy in patients with severe sepsis, the lack of pseudomonal activity and low serum levels make it a less appealing treatment in the acute phases of bacterial infection. However, after the initial treatment phase, tigecycline is an attractive antimicrobial option for deep-seated infections but use for prolonged courses via ambulatory care has historically been inhibited by the twice-daily posology. The majority of outpatient parenteral antimicrobial therapy (OPAT) antimicrobials are administered once daily to facilitate a single daily visit. Whilst multi-daily administration is possible, multiple daily visits for a patient over a prolonged period is less feasible.

The long half-life of tigecycline lends it favourably to once-daily administration with appropriately dosed tigecycline to provide adequate antimicrobial activity for OPAT patients.^5^^,^^6^ Yet there is a paucity of data to support the use of once-daily dosed tigecycline, limited by small sample size or use outside of the OPAT setting.^7^^,^^8^

To investigate the effectiveness and safety of once-daily tigecycline in an OPAT setting, we undertook a retrospective observational study in a large central London teaching hospital of patients receiving this therapy under the guidance of infectious diseases physicians, microbiologists and/or antimicrobial pharmacists.

## Methods

A retrospective case series of patients receiving once-daily IV tigecycline under the OPAT service was analysed from a single-centre NHS acute hospital, Chelsea and Westminster (London, UK).

All adult patients who received once-daily tigecycline between January 2016 and June 2018 on the OPAT service were included. Patients receiving less than 2 days of tigecycline were excluded owing to insufficient length of therapy to draw useful conclusions. Electronic patient records and pharmacy dispensing records were used to identify patients treated and to collect treatment-related outcomes. Patient demographics, including antimicrobial allergy history, comorbidities, antimicrobial indication, concurrent antimicrobial therapies, treatment duration and adverse events related to treatment were recorded.

Treatment outcomes were defined by the OPAT multidisciplinary team using the BSAC National Outcomes Registry System (NORS) at the end of each individual treatment course and grouped according to (i) complete cure: completed treatment and resolution of infection; (ii) treatment failure: either premature cessation of tigecycline owing to adverse effects or non-response of infection to antibiotic; and (iii) failure due to disease progression: disease progressed, requiring alternative aggressive treatment, although there was response in infection to the antimicrobial administered.^9^ Additional endpoints were collected, including incidence of adverse effects, serum amylase and the need for concurrent antiemetic prescription.

All data were anonymized and collated on Excel 2017. Descriptive statistics only were derived, using GraphPad^®^ (v8, 2018). Ethical consent was not required for this retrospective analysis following review by the Trust clinical governance team; it was registered as a service-evaluation project. Data were anonymized at the point of collection.

## Results

A total of 25 cases involving 24 patients were identified during the study period. The mean age was 64 (range 41–85) years. One patient received tigecycline on two separate OPAT referrals. Patients received tigecycline as OPAT for a median of 18 (range 2–117) days for a variety of infections, most commonly bone, joint or diabetic foot infections or intra-abdominal infections (Table 1), with a total of 747 bed days saved. Patients treated with tigecycline for polymicrobial infections or to target particularly resistant organisms was common [17/24 patients (71%)]. Tigecycline was often prescribed following treatment failures with other empirical regimens or in refractory disease. One patient received prolonged suppressive treatment with tigecycline despite known tigecycline-resistant pathogens and concurrent pseudomonal infections for symptom control. One patient required concurrent antibacterial therapy (ciprofloxacin) while five patients received concurrent antifungal treatment. The treatment outcomes overall, and by clinical indication, are noted in Table 1.

**Table:** Table 1. Utility of once-daily tigecycline administration in an OPAT setting, London, UK

	No. of cases (%)
Indication	
bone and joint/diabetic foot infection	8 (32)
intra-abdominal infection	7 (28)
gynaecological/urological infection	4 (16)
hepatobiliary infection	3 (12)
surgical site infection	2 (8)
other	1 (4)
Microbiology results	
Enterobacterales	16 (64)
ESBL resistance	13 (52)
NDM resistance	1 (4)
*Enterococcus* spp.	7 (28)
GRE resistance	4 (16)
*Staphylococcus aureus*	2 (8)
polymicrobial	10 (40)
no positive growth	4 (16)
Successful treatment outcomes by indication	
bone and joint/diabetic foot infection	6 (75)
intra-abdominal infection	6 (86)
gynaecological/urological infection	3 (75)
hepatobiliary infection	3 (100)
surgical site infection	1 (50)
other	0 (0)
Overall treatment outcomes^a^	
success	19 (76)
failure due to drug-related adverse reaction	3 (12)
failure due to disease progression	3 (12)

a Defined according to BSAC NORS for OPAT.

Treatment success, as defined by the OPAT team, was 76% (19/25) of cases; three cases were stopped due to drug-related adverse events and three cases were associated with clinical treatment failure.

Treatment failure Case 1 was due to disease progression. A polymorbid end-stage diabetic with refractory diabetic foot infections was stabilized on tigecycline for 3.5 months of OPAT despite presence of pan-resistant *Proteus* spp., *Klebsiella* spp. and *Pseudomonas* organisms at the site of infection. Tigecycline was continued until the patient consented to amputation as definitive cure. Treatment failure Case 2 required surgical resection of a prostate abscess following failure to respond to tigecycline and subsequently a carbapenem. Treatment failure Case 3 was changed from tigecycline following failure to treat aortic root infection; no pathogen was identified and the patient has subsequently failed to respond to long-term cephalosporin and carbapenem therapy.

All patients were counselled on the potential risk of nausea with the once-daily preparation, with eight patients (32%) reporting nausea or vomiting on subsequent weekly questioning by the OPAT team. Of these eight patients, five were managed with antiemetics successfully and treatment was continued. One patient’s nausea responded to slower IV administration (60 min rather than 30 min). Two patients required cessation of tigecycline therapy due to persistent nausea and lethargy despite antiemetic provision.

A delayed rash (5 weeks into therapy) developed in one patient; this was not rechallenged and persisted with subsequent β-lactam therapy and thus not thought to be tigecycline related. Serum amylase was measured in all patients on prolonged tigecycline treatment (>10 days) due to concerns regarding drug-induced pancreatitis. Transient increases in amylase were evident in three patients; two patients were continued on therapy as the amylase did not exceed the 2-fold upper normal laboratory limit increase (>180 IU/L) or develop any signs of drug pancreatitis. Amylase exceeding this (up to 190 IU/L) was identified in one patient in Week 1 of therapy; treatment was continued despite this, with follow-up results showing improvement at Week 2 (156 IU/L). Treatment was later stopped due to nausea and lethargy thought to be unrelated to amylase level.

## Discussion

In our selected patient population, tigecycline was effective even when administered once daily to facilitate outpatient care. A high level of treatment success (76%) was evident despite the complex nature of the patients ambulated.

Concerns over clinical effectiveness exist with tigecycline following FDA alerts highlighting treatment failures in acute bacterial infections when compared with β-lactam therapy.^10^ In this study, patients treated with IV tigecycline were carefully selected by infectious diseases consultants and antimicrobial pharmacists. Prior to consideration for OPAT, patients who met criteria for sepsis received concurrent therapy (often an aminoglycoside) as an inpatient until there was evidence of clinical improvement and sterile blood cultures confirmed. The rationale for tigecycline treatment was primarily due to the presence of polymicrobial infections, MDR organisms and/or previous antimicrobial allergies. Prescribing for off-licence indications including diabetic foot infections and bone and joint infections was common, with good success, and builds on evidence that tigecycline provides good bone penetration, exceeding the MIC for susceptible isolates.^11^

The most common adverse event from once-daily tigecycline is nausea and vomiting (up to a third of cases) but this is manageable with antiemetics and slowing the rate of infusion. Tigecycline-associated pancreatic complications were not identified in this study.

In conclusion, with a broad spectrum of activity, including MRSA, GRE, ESBL and CRO coverage, tigecycline administered once daily offers a valuable option for effective treatment of a variety of chronic infections in OPAT.

## Supplementary Material

dlz085_Supplementary_Data

## Availability of data and materials

The datasets analysed during the current study and further details on gaining access to the intervention reported within this study are available from the first author (stephen.hughes2@chelwest.nhs.uk) on reasonable request, as long as this meets local ethics and research governance criteria.

## Transparency declarations

L.S.P.M. has consulted for bioMérieux (2013), DNAelectronics (2015), Dairy Crest (2017–2018), received speaker fees from Profile Pharma (2018) and Pfizer (2018-2019), received research grants from the NIHR (2013–2019), Leo Pharma (2016), and CW+ Charity, and received educational support from Eumedica (2016–2017). N.M. has consulted for Pfizer (2019) and received educational support from Eumedica (2015) and Baxter (2017). S.H. has received educational support from Baxter (2017). B.B. has received educational support from Bayer plc and Bristol-Myers Squibb-Pfizer. All other authors: none to declare.
